# *Anaerobutyricum soehngenii* Reduces Hepatic Lipogenic Pathways and Increases Intestinal Gluconeogenic Gene Expression in Metabolic-Dysfunction-Associated Steatotic Liver Disease (MASLD) Mice

**DOI:** 10.3390/ijms25063481

**Published:** 2024-03-20

**Authors:** Anne Linde Mak, Quinten J. J. Augustijn, Clément J. F. Heymann, Stefan Havik, Xanthe Verdoes, Melany Rios-Morales, Laura A. Bosmans, Joanne Verheij, Abraham S. Meijnikman, Patrick A. de Jonge, Hilde Herrema, Willem M. de Vos, Max Nieuwdorp, Aldo Grefhorst, Adriaan G. Holleboom

**Affiliations:** 1 Department of (Experimental) Vascular Medicine, Amsterdam University Medical Centers, Location AMC, 1105 AZ Amsterdam, The Netherlands; a.l.mak@amsterdamumc.nl (A.L.M.); q.j.augustijn@amsterdamumc.nl (Q.J.J.A.); clemheymann@orange.fr (C.J.F.H.); s.r.havik@amsterdamumc.nl (S.H.); x.verdoes@amsterdamumc.nl (X.V.); m.riosmorales@amsterdamumc.nl (M.R.-M.); l.a.bosmans@amsterdamumc.nl (L.A.B.); a.s.meijnikman@amsterdamumc.nl (A.S.M.); p.a.dejonge@amsterdamumc.nl (P.A.d.J.); h.herrema@amsterdamumc.nl (H.H.); m.nieuwdorp@amsterdamumc.nl (M.N.); a.g.holleboom@amsterdamumc.nl (A.G.H.); 2 Department of Pathology, Amsterdam University Medical Centers, Location AMC, 1105 AZ Amsterdam, The Netherlands; j.verheij@amsterdamumc.nl; 3 Caelus Health, 1105 BP Amsterdam, The Netherlands; willem.devos@wur.nl; 4 Laboratory of Microbiology, Wageningen University & Research, 6708 WE Wageningen, The Netherlands; 5 Human Microbiome Research Program, Faculty of Medicine, University of Helsinki, FI-00014 Helsinki, Finland

**Keywords:** MASLD, gut microbiome, short-chain fatty acids, mice

## Abstract

Metabolic-dysfunction-associated steatotic liver disease (MASLD) is a growing health problem for which no therapy exists to date. The modulation of the gut microbiome may have treatment potential for MASLD. Here, we investigated *Anaerobutyricum soehngenii*, a butyrate-producing anaerobic bacterium with beneficial effects in metabolic syndrome, in a diet-induced MASLD mouse model. Male C57BL/6J mice received a Western-type high-fat diet and water with 15% fructose (WDF) to induce MASLD and were gavaged with *A. soehngenii* (10^8^ or 10^9^ colony-forming units (CFU) 3 times per week) or a placebo for 6 weeks. The *A. soehngenii* gavage increased the cecal butyrate concentrations. Although there was no effect on histological MASLD scores, *A. soehngenii* improved the glycemic response to insulin. In the liver, the WDF-associated altered expression of three genes relevant to the MASLD pathophysiology was reversed upon treatment with *A. soehngenii*: Lipin-1 (*Lpin1*), insulin-like growth factor binding protein 1 (*Igfbp1*) and Interleukin 1 Receptor Type 1 (*Il1r1*). *A. soehngenii* administration also increased the intestinal expression of gluconeogenesis and fructolysis genes. Although these effects did not translate into significant histological improvements in MASLD, these results provide a basis for combined gut microbial approaches to induce histological improvements in MASLD.

## 1. Introduction

The composition of the microbiota in the gut relates to the metabolic health of the host [1]. The gut microbiota interacts with passing nutrients in order to facilitate the digestion of complex fibers from the diet. During this digestive process, gut microbes can produce and secrete metabolites such as short-chain fatty acids (SCFAs) or ethanol [2]. In a well-balanced and diverse gut microbiome, the bacteria are in symbiosis with their human host, receiving nutrients from the diet and secreting potentially beneficial metabolites into the host’s circulation [3,4,5]. In multiple disease states, e.g., cardiovascular diseases, obesity, and gastrointestinal disorders, the composition of the gut microbiota is altered, resulting in reduced species diversity and the expanded abundance of pathobionts that produce metabolites unfavorable to host health [5,6]. Such metabolites are carried through the portal vein to the liver, where they are first detected and metabolized before potentially affecting the whole-body physiology.

Metabolic-dysfunction-associated steatotic liver disease (MASLD) is an emerging health problem in which various aspects of the hepatic metabolism are disturbed. MASLD is a rather recent term, replacing non-alcoholic fatty liver disease (NAFLD) [7]. Importantly, in human studies, subjects that were diagnosed as having NAFLD will now be diagnosed with MASLD [8]; hence, the term MASLD is also used for NAFLD. Peripheral insulin resistance is likely key in MASLD development since it leads to the increased flux of free fatty acids (FFAs) from the adipose tissue to the liver [9]. In addition, high blood insulin levels also increase hepatic FFA formation by stimulating de novo lipogenesis [10]. Together, these pathways result in the excessive accumulation of triglycerides (TG) in the lipid droplets within hepatocytes, a defining histological aspect of MASLD [11]. The enhanced mitochondrial β-oxidation of these lipids causes the formation of reactive oxygen species and oxidative stress, which play a part in the impairment of mitochondrial function that occurs in advanced MASLD [12]. Gradual liver injury occurs when inflammatory pathways are activated, as seen in metabolic-dysfunction-associated steatohepatitis (MASH), characterized by lobular inflammation and hepatocyte ballooning. As a result of these processes, quiescent hepatic stellate cells become activated and produce excess collagen, leading to liver fibrosis [12]. Thus, MASLD encompasses a spectrum of disease states, from isolated steatosis to active disease, i.e., MASH, and chronic damage with hepatic fibrosis. Ultimately, MASLD can lead to cirrhosis and hepatocellular carcinoma. Predisposing factors for the development and progression of MASLD are a sedentary lifestyle, a Western diet high in fructose, and genetic susceptibility.

Changes in the gut microbiome have been linked to MASLD as well as insulin resistance [13], but it has proven difficult to distinguish associations from causality. One way to explore the causality of the gut microbiota on (the parameters of) MASLD is to transfer the fecal content from a healthy donor to an MASLD and/or insulin-resistant subject by fecal microbiota transplantation (FMT) [14]. When insulin-resistant individuals received an FMT from healthy donors, their insulin sensitivity improved, alongside an elevation in the abundance of *Anaerobutyricum soehngenii* (previously known as *Eubacterium hallii* [15]) in the small intestine [16], suggesting a positive effect of this bacterium on glucose metabolism. *A. soehngenii* is a Gram-positive anaerobic bacterium of the *Lachnospiraceae* family that can produce butyrate by fermenting carbohydrates in the presence of lactate and acetate [15]. Further experiments with this bacterium showed that it improved the insulin sensitivity of genetically obese *db/db* mice [17], as well as peripheral glycemic control in subjects with metabolic syndrome [18]. Recent studies with oral *A. soehngenii* administration showed that this is safe [19] and that the fecal *A. soehngenii* abundance is positively correlated with peripheral insulin sensitivity in subjects with metabolic syndrome [20].

Of interest, *A. soehngenii* treatment reduced the hepatic expression of several lipogenic genes in *db/db* mice [17], suggesting that it might have a beneficial effect on MASLD. Since *db/db* mice have an extreme metabolic phenotype that does not reflect the human liver physiology, we decided to explore the effects of *A. soehngenii* in a diet-induced mouse MASLD model to better reflect the human situation.

## 2. Results

### 2.1. WDF Feeding Time-Dependently Induces MASLD

When mice were given a high-fat, high-cholesterol diet supplemented with 15% fructose in the drinking water (WDF), they developed increased body weights, enlarged adipose tissue deposits, elevated plasma cholesterol, elevated fasting blood glucose concentrations, increased liver TG content, and the increased hepatic expression of selected inflammatory and fibrosis genes (Figure S1). The histological evaluation of the livers showed that the hepatic steatosis worsened over time in mice on the WDF diet. When evaluating the effects of WDF feeding on hepatic fatty acid profiles, we found an increase in monounsaturated fatty acids (MUFAs) and a decrease in polyunsaturated fatty acids (PUFAs) in mice on the WDF diet compared to control mice (Figure S2A). The individual lipids that contributed most to this difference were oleic acid (18:1ω9; increase from 16.9% in controls to 38.8% in 16-week WDF) and linoleic acid (18:2ω6; decrease from 20.5% in controls to 5.3% in 16-week WDF). As linoleic acid is an essential fatty acid, we calculated the triene/tetraene ratio (T/T ratio) in the liver. We found a T/T ratio elevation over time with the WDF diet (Figure S2B), which indicated an incremental essential fatty acid deficiency, although the ratios were still below the cutoff value for essential fatty acid deficiencies in serum of 0.2 [21]. We also explored hepatic mRNA expression by RNA sequencing and identified an increasing number of differently expressed genes and activated pathways in mice over time with WDF in comparison to the control mice (Figure S3). Interestingly, the WDF feeding resulted in the marked induction of the hepatic mRNA expression of the lipogenic genes *Acaca*, *Fasn*, and *Acls1* and their transcriptional regulator *Srebf1*; the gluconeogenic genes *Pck1* and *G6pc*; the fructolytic genes *Khk, Tkt*, *Aldob*, and *Tkfc*; and the *Slc2a2* gene that encodes for the glucose transporter Glut2 (Figure S4). The most prominent activated pathways after 16 weeks of WDF feeding were p53 signaling, TNF signaling, hepatocellular carcinoma, NF-κB signaling, cytokine–cytokine receptor interaction, and pathways in cancer. Moreover, the PI3K-Akt signaling pathway was most prominently upregulated throughout all time points of WDF feeding (Figure S5).

### 2.2. A. soehngenii Supplementation Increases Cecal Butyrate Levels

**Figure 1:**
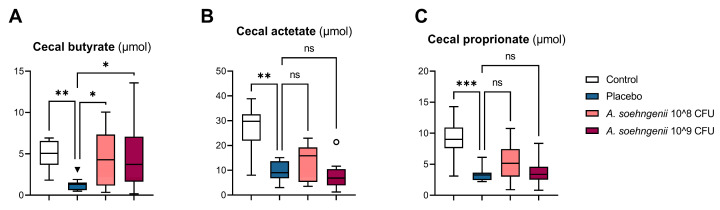
Cecal short-chain fatty acid levels after *A. soehngenii* or placebo supplementation in WDF-fed mice compared to control chow-fed mice. * *p* < 0.05; ** *p* < 0.01; *** *p* < 0.001, ns not significant, all comparisons versus placebo group. Data shown as boxplots with 25th–75th percentiles and whiskers following Tukey’s method, *n* = 8–12 mice per group.

Based on the observed changes over time with the WDF diet, we chose to apply our *A. soehngenii* intervention in mice on the WDF diet from week 10 to week 16. Since the potentially beneficial effects of *A. soehngenii* are thought to result from its butyrate-producing capacity, we measured the SCFA profiles in the ceca of the mice after 6 weeks of *A. soehngenii* supplementation. We found that the levels of all SCFAs (i.e., acetate, propionate, and butyrate) were significantly lower in WDF-fed placebo mice compared to chow-fed controls (Figure 1). In the mice supplemented with a high dose (10^9^ colony-forming units (CFU)) of *A. soehngenii*, the cecal butyrate concentration was indeed significantly higher than that in the placebo-treated mice.

### 2.3. A. soehngenii Improves Insulin Sensitivity of WDF-Fed Mice

**Figure 2:**
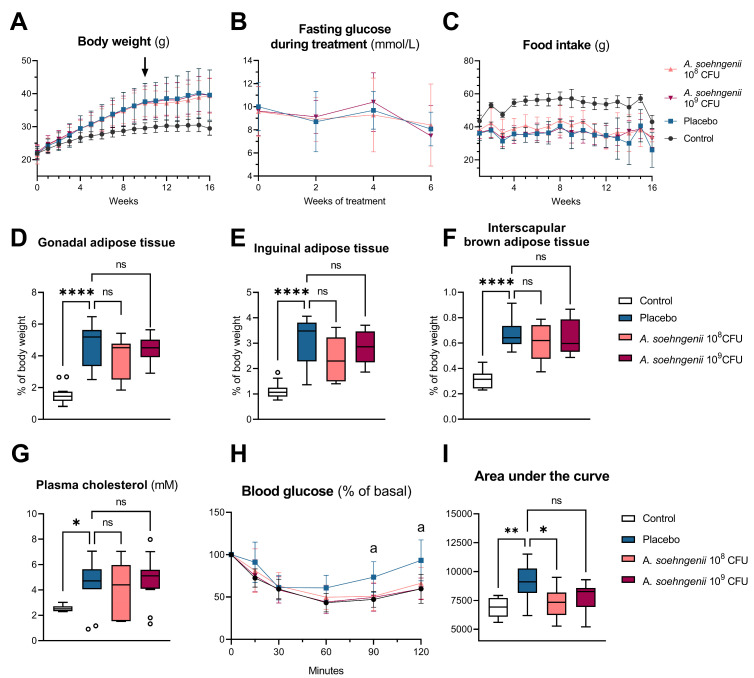
Characteristics of WDF-fed mice treated with placebo, *A. soehngenii* 10^8^ or 10^9^ CFU, and control mice fed a chow diet. (**A**) Body weight over time, with arrow depicting start of treatment; (**B**) fasting glucose during the 6-week *A. soehngenii* treatment period; (**C**) food intake over time; (**D**–**F**) adipose tissue weights as percentage of body weight; (**G**) plasma cholesterol levels; (**H**) blood glucose response after intraperitoneal administration of 0.75 IU/kg insulin, shown as the percentage of the basal glucose level at the time of administration; (**I**) area under the curve of the graph shown in (**H**). ^a^ *p* < 0.05 between placebo and *A. soehngenii* 10^9^ CFU by mixed-effect analysis; * *p* < 0.05, ** *p* < 0.01, **** *p* < 0.0001, ns not significant, all comparisons versus placebo group. Data shown as boxplots with 25th–75th percentiles and whiskers following Tukey’s method, *n* = 9–12 mice per group.

Within the 6 weeks of supplementation, *A. soehngenii* did not affect the body weight, body composition (i.e., adipose tissue mass), food intake, and plasma cholesterol concentrations (Figure 2). Although the fasting blood glucose concentrations were also not affected by *A. soehngenii*, the mice treated with *A. soehngenii* 10^8^ CFU showed a similar response to the insulin tolerance test as the control mice, which was significantly different from that of the placebo-treated mice on the WDF diet and thus reflects improved insulin sensitivity upon *A. soehngenii* treatment (Figure 2H,I).

### 2.4. A. soehngenii Does Not Affect Liver Steatosis but Restores Hepatic Expression of Multiple Genes

**Figure 3:**
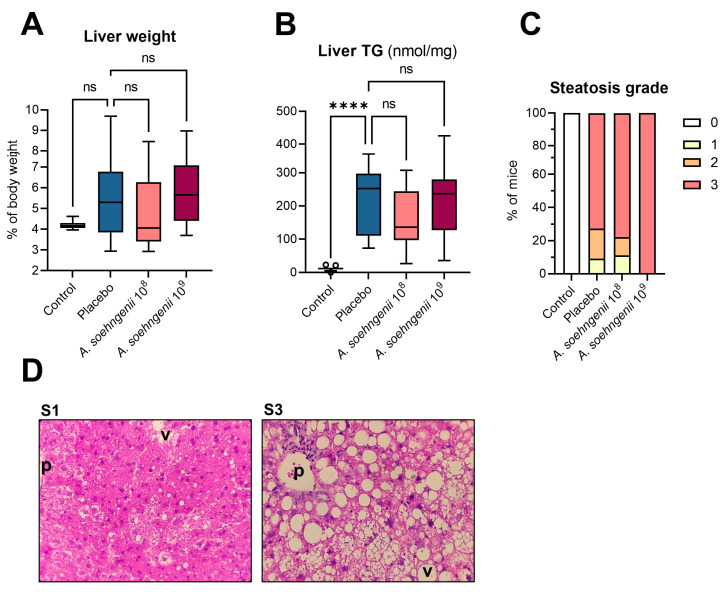
Liver characteristics of WDF-fed mice treated with placebo, *A. soehngenii* 10^8^ or 10^9^ CFU, and control mice fed a chow diet. (**A**,**B**) Mouse liver weights and triglyceride content; (**C**) scoring of the steatosis grade; (**D**) hematoxylin-and-eosin-stained examples of S1- and S3-scored livers, in which ‘p’ denotes the periportal area and ‘v’ the perivenous area. Data shown as boxplots with 25th–75th percentiles and whiskers following Tukey’s method, **** *p* < 0.0001 and ns, not significant (all comparisons versus placebo group), *n* = 9–12 mice per group.

When evaluating the liver histology MASLD scoring after *A. soehngenii* supplementation, no differences were identified in the liver TG concentrations and steatosis scoring in WDF-fed mice receiving *A. soehngenii* compared to the placebo (Figure 3).

**Figure 4:**
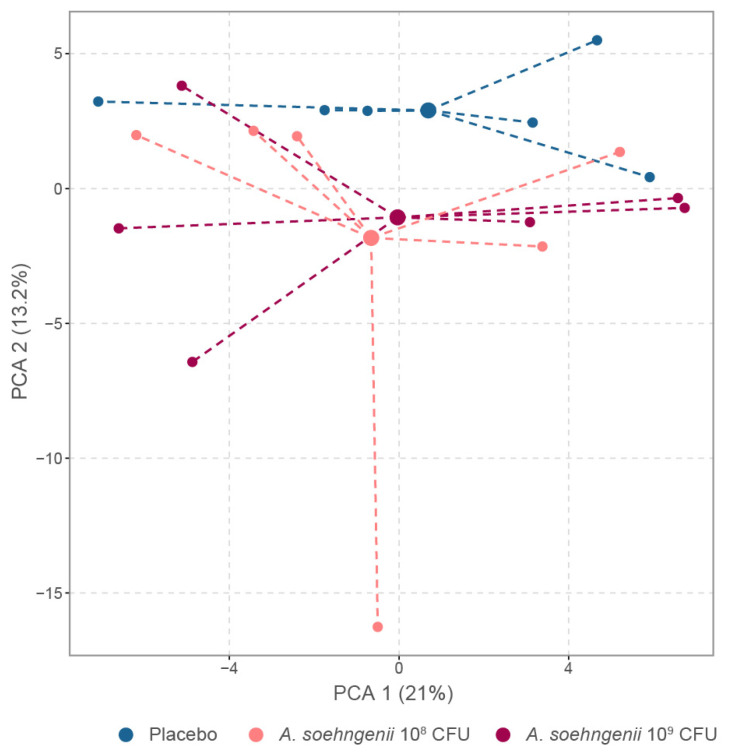
Principal component analysis of hepatic transcriptomic differences between WDF-fed mice treated with placebo, *A. soehngenii* 10^8^ or 10^9^ CFU.

In order to determine whether the *A. soehngenii* treatment had an effect on the liver, we analyzed the hepatic gene expression of the WDF-fed mice by RNA sequencing. As shown by principal component analysis (PCA), the hepatic mRNA expression profiles of the two *A. soehngenii* treatment groups were more similar to each other than that of the placebo group (Figure 4). However, when assessing genes with differential hepatic gene expression patterns, the low dose of 10^8^ CFU *A. soehngenii* had a more pronounced effect on hepatic gene expression than the high dose of 10^9^ CFU. Of the 30 genes that had differential hepatic expression levels, 19 were protein coding and mostly involved in lipid, glucose, and immune regulation, while the remainder were of unknown function (Table S1).

**Figure 5:**
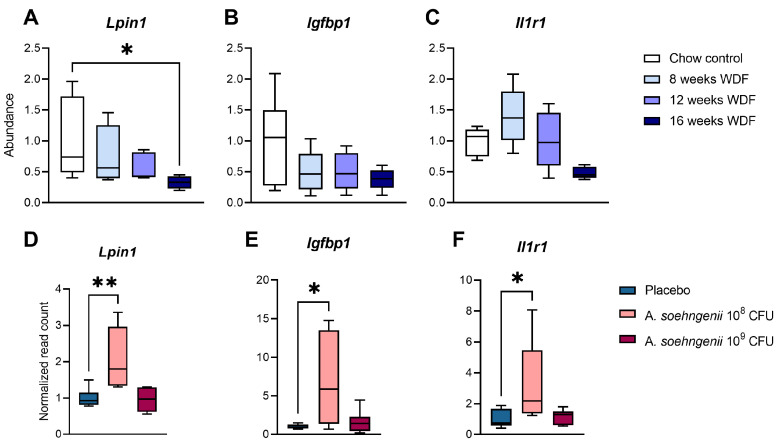
Differences in hepatic gene expression of *Lpin1*, *Igfbp1*, and *Il1r1* over time in mice on WDF diet (**A**–**C**) and between WDF-fed mice treated with placebo or *A. soehngenii* 10^8^ or 10^9^ CFU (**D**–**F**). Data shown as boxplots with 25th–75th percentiles and whiskers following Tukey’s method, *n* = 6 mice per group. Significant results versus placebo (**A**–**C**) or chow control (**D**–**F**) are shown, * *p* < 0.05; ** *p* < 0.01.

Next, we compared these latter RNA sequencing results with those of the first experiment in which mice received the WDF diet for 8, 12, or 16 weeks, and we found that the WDF-induced changes in the mRNA expression of nine genes were reversed by *A. soehngenii* (Table S2). Of these, three were significantly different between the treatment groups: Lipin-1 (*Lpin1*), insulin-like growth factor binding protein 1 (*Igfbp1*), and Interleukin 1 Receptor Type 1 (*Il1r1*) (Figure 5).

**Figure 6:**
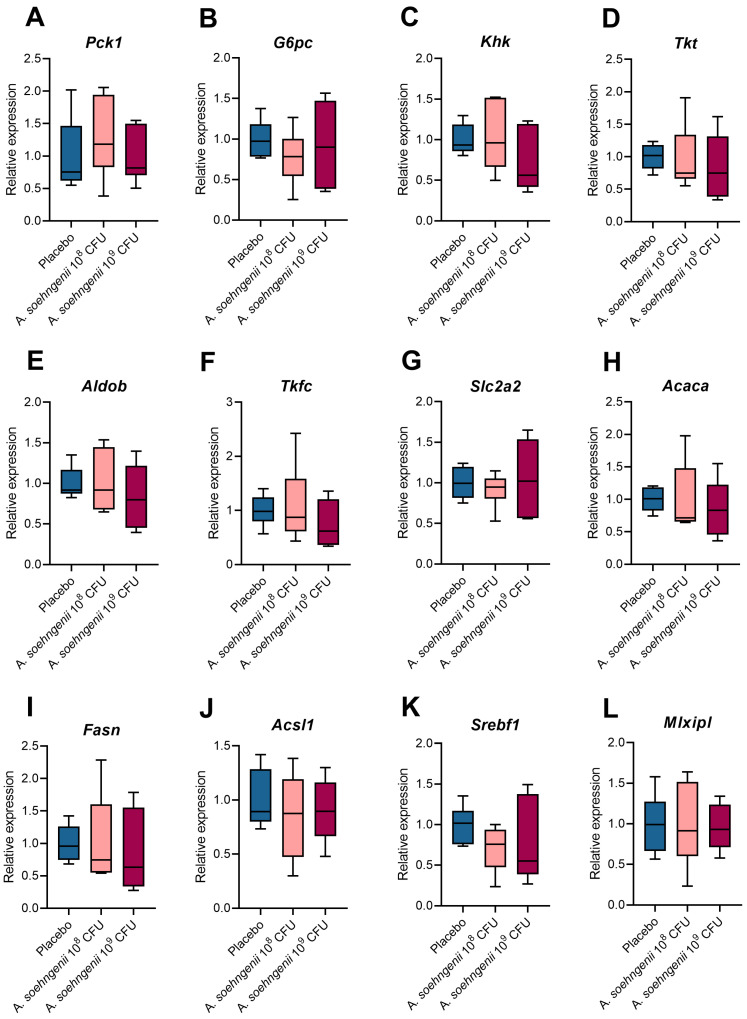
Differences in hepatic gene expression of selected gluconeogenic, fructolytic, and lipogenic genes between WDF-fed mice treated with placebo or *A. soehngenii* 10^8^ or 10^9^ CFU. Data shown as boxplots with 25th–75th percentiles and whiskers following Tukey’s method, *n* = 6 mice per group. No significant results versus placebo were found.

The expression of the selected genes that showed increased expression upon WDF (see Figure S5) was, however, not corrected by the treatment with *A. soehngenii* (Figure 6).

### 2.5. Effects of A. soehngenii Treatment on Small Intestinal Gene Expression

**Figure 7:**
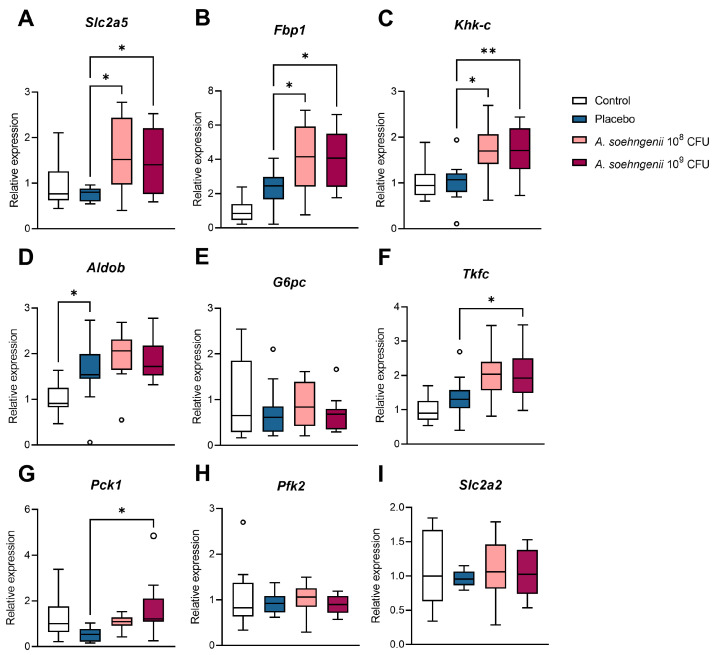
Jejunal expression of fructolytic and gluconeogenic genes. Data shown as boxplots for 25th–75th percentiles and whiskers following Tukey’s method, *n* = 9–12 mice per group. Significant results versus placebo are shown; * *p* < 0.05; ** *p* < 0.01.

Next, we measured the effect of the intervention with *A. soehngenii* and the WDF diet on the mRNA expression of genes encoding proteins involved in intestinal gluconeogenesis (IGN) and fructolysis. We found that the mRNA expression of the genes encoding for glucose transporter-5 (Glut5, encoded by *Slc2a5*), fructose bisphosphatase 1 (*Fbp1*), ketohexokinase-c (*Khk-c*), triokinase (*Tkt*), FMN cyclase (*Tkfc*), and phosphoenolpyruvate carboxykinase 1 (*Pck1*) was significantly higher in the jejunum of *A. soehngenii*-treated WDF mice than in those receiving the placebo (Figure 7). The mRNA expression of genes encoding glucose-6-phosphatase (*G6pc*), aldolase B (*Aldob*), phosphofructokinase-2 (*Pfk2*), and glucose transporter-2 (Glut2, encoded by *Slc2a2*) was not affected (Figure 7). Surprisingly, the WDF diet per se did not affect the mRNA expression of the gene encoding the fructose-specific transporter Glut5, and we therefore evaluated the effects of the WDF diet on the mRNA expression of the selected genes in the jejunum in the first experiment. In this experiment, the jejunal expression of *Slc2a5* showed an initial increase after 8 weeks of WDF, but this decreased precipitously upon prolonged WDF exposure (Figure S6). The jejunal expression of *Khk-c* and *Pck1* followed a similar pattern, while the expression of *Fbp1* and *Aldob* was higher at all time points for the WDF diet.

## 3. Discussion

Recent evidence indicates that the gut microbial compositions of patients with MASLD and its more advanced stages differ from those of healthy individuals [13,22]. This has led to the hypothesis that the gut microbiome could provide a target for the treatment of MASLD, an unmet medical need [23,24]. In this work, we indeed show that introducing a next-generation beneficial microbe, *A. soehngenii*, into mice with diet-induced MASLD can alter the response to insulin and influence hepatic and intestinal gene expression. However, no effect on histologically scored MASLD was seen in this mouse model.

In general, the mice receiving the placebo had a relatively better metabolic phenotype than the mice in the first experiment on the WDF diet. The sole difference between these two groups of mice was the oral gavage, and it is known that this might trigger a stress response that attenuates caloric intake and hence body weight gain and (metabolic) factors associated with it [25]. We therefore compared the results within and not between experiments.

We observed that WDF-fed mice treated with *A. soehngenii* had improved insulin tolerance compared to those receiving the placebo, suggesting that the intestinal presence of this bacterium can improve insulin sensitivity. This effect may be due to an increase in butyrate production by the microbe, since we saw increased cecal butyrate levels in the treatment groups. A recent systematic review reported that increased butyrate levels are associated with improved insulin sensitivity, although, importantly, butyrate was not associated with lower fasting glucose levels, which we also did not see in these mice [26]. The improved glycemic control after the administration of *A. soehngenii* is in line with findings in both diabetic *db/db* mice [16] and a human trial in which a single duodenal infusion of *A. soehngenii* in metabolic syndrome subjects was found to improve peripheral glycemic control, potentially via an increase in serum GLP-1 levels [18].

*A. soehngenii* administration also induced changes in hepatic gene expression, as detailed below. Interestingly, the differences were more pronounced when *A. soehngenii* was given as a low dose (10^8^ CFU) than as a high dose (10^9^ CFU). This finding is in accordance with a previous dose-finding study with *A. soehngenii* in which it was also shown that 10^8^ CFU was the most potent dose when compared to doses of 10^6^ and 10^10^ CFU [17]. Dose response studies with administered microbes are limited in number. Nonetheless, another example is *B. infantis*, for which the middle and not the high dose was again the most efficient in treating irritable bowel syndrome [27]. Therefore, in line with other studies, these results indicate that treatment with excessive dosages of single bacteria may lead to reduced effects. We postulate that, as a result of high dosages of single bacterial treatments, other beneficial cross-feeding bacteria may be repressed, potentially leading to a reduction in the beneficial effects of the single bacterial treatment.

To deeply characterize our model and treatment, we assessed liver transcriptomic changes over time on the WDF diet. Subsequent pathway analysis showed the induction of insulin resistance, PPAR signaling, and pathways involved in inflammation and fibrosis over time on the diet, underscoring the validity of this model for MASLD. When comparing the RNA sequencing results from the mouse livers in the first experiment with those of livers after the supplementation of *A. soehngenii*, we found that the treatment with *A. soehngenii* restored the expression of *Lpin1*, *Igfbp1*, and *Il1r1*.

The *Lpin1* gene encodes the enzyme Lipin-1 that catalyzes the penultimate step in TG synthesis. Moreover, it functions as a nuclear transcriptional coactivator to modulate the expression of other genes involved in lipid metabolism [28]. *Lpin1* expression is induced by peroxisome proliferator-activated receptor gamma (PPARgamma) coactivator 1alpha (PGC-1alpha) and selectively activates fatty acid oxidation and mitochondrial oxidative phosphorylation, whilst suppressing lipogenesis [29]. In our study, the expression of *Lpin1* declined over time with WDF feeding, whereas it increased after *A. soehngenii* administration. It has been shown that the hepatic *Lpin1* expression is diminished in insulin-resistant obese individuals, while gastric bypass surgery causes the increased expression of *Lpin1* [30]. Taken together, this suggests that the increase in *Lpin1* expression after *A. soehngenii* administration is beneficial to hepatic lipid metabolism.

IGFBP1 is involved in glucose regulation and insulin resistance and regulates hepatic lipogenesis and fatty acid beta-oxidation [31]. It has previously been found to be a marker for advanced fibrosis in MASLD [32]. However, treatment with exogenous IGFBP1 was recently shown to improve hepatic steatosis and liver TG content and reduce serum ALT and AST in MASLD mice [33]. These contrasting findings may be due to differences in function between phosphorylated and non-phosphorylated IGFBP1 [33]. In our study, the hepatic *Igfbp1* mRNA expression decreased over time when the mice were fed the WDF diet, and *A. soehngenii* supplementation increased the hepatic *Igfbp1* mRNA expression. Thus, although it is clear that Igfbp1 plays an important role in hepatic metabolism, further research is required to identify the exact function of this gene in MASLD.

*Il1r1* encodes the receptor for the Interleukin-1 family cytokines IL1α, IL1β, and IL1RA. After cytokine binding, it associates with the co-receptor IL1RAP to mediate the activation of NFκB, MAPK, and other immunoregulatory signaling pathways [34]. The gene expression of *Il1r1* was downregulated after 16 weeks of WDF feeding and rose after *A. soehngenii* treatment compared to the placebo. In contrast, previous research has shown that the hepatic knockout of *Il1r1* protected against acute liver injury in mice [35]. However, as MASLD is a slow progressive disease in which immune signaling plays an important role, one may expect IL1R1 signaling to have different effects compared to acute inflammatory liver injury, potentially explaining these opposing results. Indeed, we saw that *Il1r1* expression was firstly increased after the first 8 weeks on the diet, and only decreased after continued WDF feeding.

Despite these hepatic gene-level effects of the bacterial treatment, our data show that *A. soehngenii* as a single microbe does not lead to improvements in MASLD. Despite the within-group variation, this WDF-diet-induced MASLD model was quite severe with regard to hepatic steatosis. We chose to time this intervention before the emergence of the very severe stages of MASLD, as we expected *A. soehngenii* to be most effective in early-stage disease. Still, almost all mice on the WDF diet had >80% of hepatocytes filled with lipid droplets, and it is therefore possible that this model may have been too severe to assess the potential beneficial changes in liver steatosis by treatment with a single microbe. 

The relatively short treatment duration with *A. soehngenii* might be another reason that no effect on MASLD was observed. Moreover, *A. soehngenii* might be more potent when combined with prebiotic fiber or when administered in combination with other microbes as a probiotic cocktail. For instance, the co-administration of a lactate-producing microbe such as *Bifidobacterium adolescentis* could provide a lactate source for *A. soehngenii* to metabolize into butyrate [36]. Others have shown that metabolic cross-feeding occurs between *A. muciniphila* and *A. soehngenii* through vitamin B12 and acetate, which may also enhance the beneficial effects on host metabolism [37]. 

Previous work has shown that in the fasted state, up to 20% of glucose production may come from the intestine [38] and that intestinal gluconeogenesis (IGN) can be activated by microbiota-produced butyrate [39]. IGN has been reported as an important signal in the central enhancement of whole-body insulin sensitivity, and mice lacking intestinal G6PC, the key enzyme of IGN, develop insulin resistance despite a normal diet [40,41]. Moreover, the induction of IGN has been shown to protect against MASLD in high-fat mouse models [42]. In a human study, the infusion of *A. soehngenii* directly into the duodenum provoked marked changes in the intestinal mRNA expression of genes involved in fructose metabolism [18]. Hence, we determined the intestinal mRNA expression of genes encoding proteins involved in IGN and found that *A. soehngenii* treatment upregulated the expression of IGN-related genes *Slc2a5*, *Khk-c*, and *Tkfc*, and all three encode proteins involved in fructolysis [43]. Treatment with *A. Soehngenii* also elevated the intestinal *Fbp1* mRNA expression, which is of interest since it was reported that the FBP1 protein is activated by the short-chain fatty acid succinate [44]. We did not measure the fecal or intestinal succinate content of the mice, but it is very unlikely that this SCFA was affected by *A. Soehngenii* since this bacterium produces butyrate and not succinate [15]. 

Since the intestinal mRNA expression of typical gluconeogenic genes such as *Pck1* was not affected by the bacterial treatments, it is likely that fructolysis over IGN is induced by *A. soehngenii*, likely in response to the butyrate produced by *A. soehngenii*. The increased intestinal fructose uptake capacity would reduce the spillage of excess unmetabolized fructose to the colon [45]. This might reduce MASLD, as previous studies have suggested that most toxicity from dietary fructose is derived from its gut microbial processing into toxic plasma metabolites in the large intestine [46,47]. This could, in part, underlie the differences seen in hepatic gene expression after *A. soehngenii* administration. However, further research should be performed to confirm this hypothesis, i.e., by measuring fluxes in ingested labeled fructose.

In conclusion, we show here that the oral administration of the butyrate producer *A. soehngenii* in MASLD mice improves glycemic control, potentially via butyrate and increased IGN. *A. soehngenii* also affects hepatic lipogenic and inflammatory gene pathways, although this did not translate into a significant histological reduction in liver steatosis in this severe diet-induced MASLD model. *A. soehngenii* administration may be more beneficial to the liver when administered in the earlier stages of MASLD, potentially in combination with prebiotic fiber or other cross-feeding microbes.

## 4. Materials and Methods

### 4.1. Mouse Studies

The studies were conducted according to the guidelines of and after approval from the AMC Animal Welfare Committee under protocol number AVD11800-2016-794, in accordance with the ARRIVE guidelines.

#### 4.1.1. Experiment 1: Diet-Induced Development of MASLD

Male C57BL/6J mice were ordered from Charles River at 6 weeks of age. The mice were housed in sets of 2 per cage. After an acclimatization week, the mice were randomized by body weight and divided into 4 groups of 10 mice. Three groups were started on a high-fat and high-cholesterol diet (D12079Bi; ResearchDiets, New Brunswick, NJ, USA; composition in Table S3) and received drinking water supplemented with 15% fructose (WDF diet) [48,49]. One group of 10 mice stayed on regular chow (2916; Envigo Teklad, Indianapolis, IN, USA; composition in Table S3) as a control group. Food and water were weighed and changed weekly. Body weight and 6-*h* fasting blood glucose (FBG) were measured weekly and bi-weekly, respectively. The mice were sacrificed when they were on the WDF diet for 8, 12, or 16 weeks. On the day of sacrifice, the mice were fasted for 6 h in the morning, after which the FBG level was determined, and the mice were euthanized by cardiac puncture under isoflurane anesthesia. Blood was stored immediately at 4 °C and various tissues were dissected, weighed, and snap-frozen in liquid nitrogen or fixed in 4% paraformaldehyde (PFA) in PBS at room temperature. The distinct interscapular brown adipose tissue (BAT) deposit and the gonadal and inguinal white adipose tissue (WAT) deposits were collected as reported by Bagchi and MacDougald [50]. The small intestines were removed and divided into three equal sections. Approximately 5 mm in the center of the middle part was collected and used to determine the mRNA expression of the jejunum.

#### 4.1.2. Experiment 2: *A. soehngenii* Supplementation

Male C57BL/6J mice from Charles River, ordered at 4–5 weeks of age, were acclimatized and housed in 2 per cage. Three groups of 12 mice were started on the WDF diet at 7 weeks of age, and one group of 12 mice continued the regular chow diet as a control group. From 10 to 16 weeks on the WDF diet, the mice received a thrice-weekly gavage with frozen anaerobic *A. soehngenii* at 10^8^ or 10^9^ CFU (strain CH106, which has been shown to be non-toxic and safe [19]) in a cryoprotectant solution or only the cryoprotectant as a placebo control. The cryoprotectant consisted of anaerobic PBS + 15% sorbitol + 0.05% cysteine hydrochloride. After 5 weeks of gavage, the fasted mice were subjected to an insulin tolerance test (ITT; see below). One week after the ITT, the mice were sacrificed as mentioned above for Experiment 1. The production and viability testing of *A. soehngenii* cultures was performed as previously described [51].

#### 4.1.3. Insulin Tolerance Testing (ITT)

The mice were fasted for 4–6 h, after which the blood glucose concentration was measured in a small blood drop from the tail with a handheld ContourXT blood glucose meter. Next, 0.75 IU/kg of human insulin (NovoRapid; NovoNordisk, Alphen a.d. Rijn, The Netherlands) dissolved in a solution of 1% BSA (Sigma-Aldrich, Zwijndrecht, The Netherlands) in PBS was administered intraperitoneally. Glucose levels were recorded at various time points after insulin administration.

### 4.2. Cecal SCFA Measurement

Cecal SCFA concentrations were measured using gas chromatography–mass spectrometry (GC-MS), as described previously [52]. Briefly, samples were thawed, diluted in 500 µL of PBS, and spiked with 50 µL of 10 mM 2-ethyl butyric acid as an internal standard. After adding 10 µL of HC, samples were homogenized by bead beating (6000 rpm for 2 × 20 s) and centrifuged (20 min, 15,000× *g*, 4 °C). Supernatants were transferred to glass vials and a spatula tip of solid NaCl was added. SCFAs were extracted in 2 mL diethylether and derivatized with N-tert-butyldimethylsilyl-N-methyltrifluoroacetamide (MTBSTFA) overnight. An Agilent 5975C series GC-MS machine (Agilent Technologies, Santa Clara, CA, USA) equipped with a ZB-1 column (Phenomenex, Torrance, CA, USA) was used for mass spectrometry analysis. The mass isotopolog spectra of the ([M-57]+) fragment of the t-BDMS derivatives of acetate (*m*/*z* 117), propionate (*m*/*z* 131), and butyrate (*m*/*z* 145) were monitored. The total amount of each SCFA by weight of the cecum was calculated.

### 4.3. Liver Histology Analyses

Formalin-fixed-paraffin-embedded liver slides were stained with hematoxylin and eosin. An experienced liver pathologist (JV), blinded to the grouping, scored the histology slides on the presence of steatosis according to the MASH-CRN criteria [53,54].

### 4.4. Hepatic Lipid Quantification

Mouse liver samples (6 per group) were randomly selected for hepatic lipid quantification. First, 50–150 mg pieces of frozen liver were used to produce homogenates in ice-cold PBS. Lipid extraction was performed following the method of Bligh and Dyer [55] to determine the hepatic TG content using the DiaSys Triglycerides FS 10’ kit (DiaSys Diagnostic Systems, Holzheim, Germany).

### 4.5. Hepatic Lipid Profiling

Frozen liver pieces were homogenized in ice-cold PBS, after which the hepatic fatty acid profiles were analyzed as previously described [56].

### 4.6. RNA Extraction and Quantitative RT-PCR Analyses

Total RNA was isolated from frozen tissues using TriPure Isolation Reagent (Roche Applied Sciences, Almere, The Netherlands), according to the manufacturer’s instructions. Reverse transcription was performed using a cDNA synthesis kit (SensiFAST cDNA synthesis kit, Bioline, London, UK), according to the manufacturer’s instructions. Quantitative RT-PCR was performed using SensiFAST SYBRgreen (Bioloine, London, UK) with a CFX384 Real-Time PCR System (Bio-Rad Laboratories, Hercules, CA, USA). Primer sequences are listed in Table S4. The expression of each gene was reported in arbitrary units after normalization to the average expression level of the reference genes using the 2^−ΔΔCt^ method for liver and 2^−ΔCt^ for intestinal tissue.

### 4.7. RNA Sequencing

RNA samples from mouse livers (6 per group, as for hepatic lipid quantification) were randomly selected for RNA sequencing analysis. In total, 500 ng RNA per sample was used for library preparation using the KAPA mRNA hyperprep protocol (Roche Applied Sciences, Almere, The Netherlands). Samples were sequenced using an Illumina Novaseq 6000 platform (Illumina, San Diego, CA, USA), which yielded 32.1 ± 22.4 M reads per sample (median: 23.5 M reads), at the Core Facility Genomics of Amsterdam UMC. Raw sequencing reads were trimmed for adapter sequences and quality filtered using fastp v 0.23.2 [57]. Quality-filtered reads were then mapped against the mouse transcriptome GRCm38 using Kallisto v0.48.0 [58].

Differential gene expression was determined using the DESeq2 v1.36.0 package in R [59], while the pathway analysis of differentially expressed genes was performed using the EnrichR R package v3.1 [60] against the KEGG pathway database. Finally, linear models were created using the MaAsLin2 v1.1.0 package [61] in R. The R analyses used R version 4.2.1. Where appropriate, statistical tests were adjusted for multiple testing with the Benjamini–Hochberg method.

### 4.8. Statistical Analyses

Figures were rendered in GraphPad Prism 9 or in R version 4.2.1. Outliers were identified using the ROUT method with Q = 1% and excluded if appropriate. The normality of the data distribution was analyzed using Shapiro–Wilk tests. The statistical significance of normally distributed data was tested using (repeated-measures) ANOVA and that of non-normally distributed data was tested using Mann–Whitney U or Kruskal–Wallis tests. If significant, Dunnett’s or Dunn’s tests were subsequently used to compare specific groups. Differences in response during ITTs were evaluated by mixed-effect analysis with multiple comparisons, as well as by one-way ANOVA of the area under the curve (AUC).

For principal component analysis (PCA), only genes that had at least 0.1% relative abundance in at least 1 individual were selected, and, of these genes, the raw read counts were used for a centered log-ratio transformation to offset data compositionality. CLR-transformed data were used to create a Euclidean distance matrix and to calculate significance with the PERMANOVA test (999 permutations). The Euclidean distance matrix was used to perform the PCA with the ordinate function in the R package phyloseq version 1.44.0, with the CLR-transformed data as input, method RDS, distance Euclidean. In the PCA plot, the variance of the first two components is noted on the axis (axis 1 = 21.1%, axis 2 = 13%).

## Data Availability

The data that support the findings of this study are available from the corresponding author upon reasonable request.

## Supplementary Materials

The following supporting information can be downloaded at: https://www.mdpi.com/article/10.3390/ijms25063481/s1.

## References

[1] Fan Y., Pedersen O. (2021). Gut microbiota in human metabolic health and disease. Nat. Rev. Microbiol..

[2] Sittipo P., Shim J.W., Lee Y.K. (2019). Microbial metabolites determine host health and the status of some diseases. Int. J. Mol. Sci..

[3] Adak A., Khan M.R. (2019). An insight into gut microbiota and its functionalities. Cell. Mol. Life Sci..

[4] Makki K., Deehan E.C., Walter J., Bäckhed F. (2018). The Impact of Dietary Fiber on Gut Microbiota in Host Health and Disease. Cell Host Microbe.

[5] Wang Z., Zhao Y. (2018). Gut microbiota derived metabolites in cardiovascular health and disease. Protein Cell.

[6] Hills R.D.J., Pontefract B.A., Mishcon H.R., Black C.A., Sutton S.C., Theberge C.R. (2019). Gut Microbiome: Profound Implications for Diet and Disease. Nutrients.

[7] Rinella M.E., Lazarus J.V., Ratziu V., Francque S.M., Sanyal A.J., Kanwal F., Romero D., Abdelmalek M.F., Anstee Q.M., Arab J.P. (2023). A multisociety Delphi consensus statement on new fatty liver disease nomenclature. Hepatology.

[8] Chen L., Tao X., Zeng M., Mi Y., Xu L. (2024). Clinical and histological features under different nomenclatures of fatty liver disease: NAFLD, MAFLD, MASLD and MetALD. J. Hepatol..

[9] Fabbrini E., Mohammed B.S., Magkos F., Korenblat K.M., Patterson B.W., Klein S. (2008). Alterations in adipose tissue and hepatic lipid kinetics in obese men and women with nonalcoholic fatty liver disease. Gastroenterology.

[10] Ameer F., Scandiuzzi L., Hasnain S., Kalbacher H., Zaidi N. (2014). De novo lipogenesis in health and disease. Metabolism.

[11] Ruissen M.M., Mak A.L., Beuers U., Tushuizen M.E., Holleboom A.G. (2010). Non-alcoholic fatty liver disease: A multidisciplinary approach towards a cardiometabolic liver disease. Eur. J. Endocrinol..

[12] Bessone F., Razori M.V., Roma M.G. (2019). Molecular pathways of nonalcoholic fatty liver disease development and progression. Cell Mol. Life Sci..

[13] Effenberger M., Grander C., Grabherr F., Tilg H. (2023). Nonalcoholic Fatty Liver Disease and the Intestinal Microbiome: An Inseparable Link. J. Clin. Transl. Hepatol..

[14] Aron-Wisnewsky J., Warmbrunn M.V., Nieuwdorp M., Clément K. (2020). Nonalcoholic Fatty Liver Disease: Modulating Gut Microbiota to Improve Severity?. Gastroenterology.

[15] Shetty S.A., Zuffa S., Bui T.P.N., Aalvink S., Smidt H., De Vos W.M. (2018). Reclassification of eubacterium hallii as Anaerobutyricum hallii gen. nov.; comb. nov.; and description of Anaerobutyricum soehngenii sp. nov.; a butyrate and propionate-producing bacterium from infant faeces. Int. J. Syst. Evol. Microbiol..

[16] Vrieze A., Van Nood E., Holleman F., Salojärvi J., Kootte R.S., Bartelsman J.F., Dallinga-Thie G.M., Ackermans M.T., Serlie M.J., Oozeer R. (2012). Transfer of intestinal microbiota from lean donors increases insulin sensitivity in individuals with metabolic syndrome. Gastroenterology.

[17] Udayappan S., Manneras-Holm L., Chaplin-Scott A., Belzer C., Herrema H., Dallinga-Thie G.M., Duncan S.H., Stroes E.S.G., Groen A.K., Flint H.J. (2016). Oral treatment with Eubacterium hallii improves insulin sensitivity in db/db mice. NPJ Biofilms Microbiomes.

[18] Koopen A., Witjes J., Wortelboer K., Majait S., Prodan A., Levin E., Herrema H., Winkelmeijer M., Aalvink S., Bergman J.J.G.H.M. (2021). Duodenal Anaerobutyricum soehngenii infusion stimulates GLP-1 production.; ameliorates glycaemic control and beneficially shapes the duodenal transcriptome in metabolic syndrome subjects: A randomised double-blind placebo-controlled cross-over study. Gut.

[19] Seegers J.F.M.L., Gül I.S., Hofkens S., Brosel S., Schreib G., Brenke J., Donath C., de Vos W.M. (2022). Toxicological safety evaluation of live Anaerobutyricum soehngenii strain CH106. J. Appl. Toxicol..

[20] Gilijamse P.W., Hartstra A.V., Levin E., Wortelboer K., Serlie M.J., Ackermans M.T., Herrema H., Nederveen A.J., Imangaliyev S., Aalvink S. (2020). Treatment with Anaerobutyricum soehngenii: A pilot study of safety and dose–response effects on glucose metabolism in human subjects with metabolic syndrome. NPJ Biofilms Microbiomes.

[21] Werner A., Minich D.M., Havinga R., Bloks V., Van Goor H., Kuipers F., Verkade H.J. (2002). Fat malabsorption in essential fatty acid-deficient mice is not due to impaired bile formation. Am. J. Physiol. Gastrointest. Liver Physiol..

[22] Leung H., Long X., Ni Y., Qian L., Nychas E., Siliceo S.L., Pohl D., Hanhineva K., Liu Y., Xu A. (2022). Risk assessment with gut microbiome and metabolite markers in NAFLD development. Sci. Transl. Med..

[23] Koopman N., Molinaro A., Nieuwdorp M., Holleboom A.G. (2019). Review article: Can bugs be drugs? The potential of probiotics and prebiotics as treatment for non-alcoholic fatty liver disease. Aliment. Pharmacol. Ther..

[24] Chen J., Vitetta L. (2020). Gut Microbiota Metabolites in NAFLD Pathogenesis and Therapeutic Implications. Int. J. Mol. Sci..

[25] de Meijer V.E., Le H.D., Meisel J.A., Puder M. (2010). Repetitive orogastric gavage affects the phenotype of diet-induced obese mice. Physiol. Behav..

[26] Pham N.H.T., Joglekar M.V., Wong W.K.M., Nassif N.T., Simpson A.M., Hardikar A.A. (2023). Short-chain fatty acids and insulin sensitivity: A systematic review and meta-analysis. Nutr. Rev..

[27] Whorwell P.J., Altringer L., Morel J., Bond Y., Charbonneau D., O’Mahony L., Kiely B., Shanahan F., Quigley E.M. (2006). Efficacy of an encapsulated probiotic Bifidobacterium infantis 35624 in women with irritable bowel syndrome. Am. J. Gastroenterol..

[28] Reue K., Dwyer J.R. (2009). Lipin proteins and metabolic homeostasis. J. Lipid Res..

[29] Finck B.N., Gropler M.C., Chen Z., Leone T.C., Croce M.A., Harris T.E., Lawrence J.C., Kelly D.P. (2006). Lipin 1 is an inducible amplifier of the hepatic PGC-1alpha/PPARalpha regulatory pathway. Cell Metab..

[30] Croce M.A., Eagon J.C., LaRiviere L.L., Korenblat K.M., Klein S., Finck B.N. (2007). Hepatic lipin 1beta expression is diminished in insulin-resistant obese subjects and is reactivated by marked weight loss. Diabetes.

[31] Lee P.D., Conover C.A., Powell D.R. (1993). Regulation and function of insulin-like growth factor-binding protein-1. Proc. Soc. Exp. Biol. Med..

[32] Hagström H., Stål P., Hultcrantz R., Brismar K., Ansurudeen I. (2017). IGFBP-1 and IGF-I as markers for advanced fibrosis in NAFLD—A pilot study. Scand. J. Gastroenterol..

[33] Pan J., Cen L., Zhou T., Yu M., Chen X., Jiang W., Li Y., Yu C., Shen Z. (2021). Insulin-like growth factor binding protein 1 ameliorates lipid accumulation and inflammation in nonalcoholic fatty liver disease. J. Gastroenterol. Hepatol..

[34] Petrasek J., Bala S., Csak T., Lippai D., Kodys K., Menashy V., Barrieau M., Min S.Y., Kurt-Jones E.A., Szabo G. (2012). IL-1 receptor antagonist ameliorates inflammasome-dependent alcoholic steatohepatitis in mice. J. Clin. Investig..

[35] Gehrke N., Hövelmeyer N., Waisman A., Straub B.K., Weinmann-Menke J., Wörns M.A., Galle P.R., Schattenberg J.M. (2018). Hepatocyte-specific deletion of IL1-RI attenuates liver injury by blocking IL-1 driven autoinflammation. J. Hepatol..

[36] Belenguer A., Duncan S.H., Calder A.G., Holtrop G., Louis P., Lobley G.E., Flint H.J. (2006). Two routes of metabolic cross-feeding between Bifidobacterium adolescentis and butyrate-producing anaerobes from the human gut. Appl. Environ. Microbiol..

[37] Belzer C., Chia L.W., Aalvink S., Chamlagain B., Piironen V., Knol J., de Vos W.M. (2017). Microbial Metabolic Networks at the Mucus Layer Lead to Diet-Independent Butyrate and Vitamin B(12) Production by Intestinal Symbionts. mBio.

[38] Gautier-Stein A., Rajas F., Mithieux G. (2021). Intestinal gluconeogenesis and protein diet: Future directions. Proc. Nutr. Soc..

[39] De Vadder F., Kovatcheva-Datchary P., Goncalves D., Vinera J., Zitoun C., Duchampt A., Bäckhed F., Mithieux G. (2014). Microbiota-generated metabolites promote metabolic benefits via gut-brain neural circuits. Cell.

[40] Mithieux G., Andreelli F., Magnan C. (2009). Intestinal gluconeogenesis: Key signal of central control of energy and glucose homeostasis. Curr. Opin. Clin. Nutr. Metab. Care.

[41] Soty M., Penhoat A., Amigo-Correig M., Vinera J., Sardella A., Vullin-Bouilloux F., Zitoun C., Houberdon I., Mithieux G. (2015). A gut-brain neural circuit controlled by intestinal gluconeogenesis is crucial in metabolic health. Mol. Metab..

[42] Vily-Petit J., Soty-Roca M., Silva M., Raffin M., Gautier-Stein A., Rajas F., Mithieux G. (2020). Intestinal gluconeogenesis prevents obesity-linked liver steatosis and non-alcoholic fatty liver disease. Gut.

[43] Lee H.-J., Cha J.-Y. (2018). Recent insights into the role of ChREBP in intestinal fructose absorption and metabolism. BMB Rep..

[44] Wang K., Liao M., Zhou N., Bao L., Ma K., Zheng Z., Wang Y., Liu C., Wang W., Wang J. (2019). Parabacteroides distasonis Alleviates Obesity and Metabolic Dysfunctions via Production of Succinate and Secondary Bile Acids. Cell Rep..

[45] Jang C., Hui S., Lu W., Cowan A.J., Morscher R.J., Lee G., Liu W., Tesz G.J., Birnbaum M.J., Rabinowitz J.D. (2018). The Small Intestine Converts Dietary Fructose into Glucose and Organic Acids. Cell Metab..

[46] Softic S., Gupta M.K., Wang G.-X., Fujisaka S., O’Neill B.T., Rao T.N., Willoughby J., Harbison C., Fitzgerald K., Ilkayeva O. (2017). Divergent effects of glucose and fructose on hepatic lipogenesis and insulin signaling. J. Clin. Investig..

[47] Zhao S., Jang C., Liu J., Uehara K., Gilbert M., Izzo L., Zeng X., Trefely S., Fernandez S., Carrer A. (2020). Dietary fructose feeds hepatic lipogenesis via microbiota-derived acetate. Nature.

[48] Widjaja A.A., Singh B.K., Adami E., Viswanathan S., Dong J., D’Agostino G.A., Ng B., Lim W.W., Tan J., Paleja B.S. (2019). Inhibiting Interleukin 11 Signaling Reduces Hepatocyte Death and Liver Fibrosis.; Inflammation.; and Steatosis in Mouse Models of Nonalcoholic Steatohepatitis. Gastroenterology.

[49] Sangüesa G., Baena M., Hutter N., Montañés J.C., Sánchez R.M., Roglans N., Laguna J.C., Alegret M. (2017). The Addition of Liquid Fructose to a Western-Type Diet in LDL-R(-/-) Mice Induces Liver Inflammation and Fibrogenesis Markers without Disrupting Insulin Receptor Signalling after an Insulin Challenge. Nutrients.

[50] Bagchi D.P., MacDougald O.A. (2019). Identification and Dissection of Diverse Mouse Adipose Depots. J. Vis. Exp..

[51] Wortelboer K., Koopen A.M., Herrema H., de Vos W.M., Nieuwdorp M., Kemper E.M. (2022). From fecal microbiota transplantation toward next-generation beneficial microbes: The case of Anaerobutyricum soehngenii. Front. Med..

[52] Rios-Morales M., van Trijp M.P.H., Rösch C., An R., Boer T., Gerding A., de Ruiter N., Koehorst M., Heiner-Fokkema M.R., Schols H.A. (2021). A toolbox for the comprehensive analysis of small volume human intestinal samples that can be used with gastrointestinal sampling capsules. Sci. Rep..

[53] Brunt E.M., Kleiner D.E., Wilson L.A., Belt P., Neuschwander-Tetri B.A. (2011). Nonalcoholic fatty liver disease (NAFLD) activity score and the histopathologic diagnosis in NAFLD: Distinct clinicopathologic meanings. Hepatology.

[54] Kleiner D.E., Brunt E.M., Van Natta M., Behling C., Contos M.J., Cummings O.W., Ferrell L.D., Liu Y.C., Torbenson M.S., Unalp-Arida A. (2005). Design and validation of a histological scoring system for nonalcoholic fatty liver disease. Hepatology.

[55] Bligh E.G., Dyer W.J. (1959). A rapid method of total lipid extraction and purification. Can. J. Biochem. Physiol..

[56] Muskiet F.A.J., van Doormaal J.J., Martini I.A., Wolthers B.G., van der Slik W. (1983). Capillary gas chromatographic profiling of total long-chain fatty acids cholesterol in biological materials. J. Chromatogr. B Biomed. Sci. Appl..

[57] Chen S., Zhou Y., Chen Y., Gu J. (2018). Fastp: An ultra-fast all-in-one FASTQ preprocessor. Bioinformatics.

[58] Bray N.L., Pimentel H., Melsted P., Pachter L. (2016). Near-optimal probabilistic RNA-seq quantification. Nat. Biotechnol..

[59] Love M.I., Huber W., Anders S. (2014). Moderated estimation of fold change and dispersion for RNA-seq data with DESeq2. Genome Biol..

[60] Chen E.Y., Tan C.M., Kou Y., Duan Q., Wang Z., Meirelles G.V., Clark N.R., Ma’ayan A. (2013). Enrichr: Interactive and collaborative HTML5 gene list enrichment analysis tool. BMC Bioinform..

[61] Mallick H., Rahnavard A., McIver L.J., Ma S., Zhang Y., Nguyen L.H., Tickle T.L., Weingart G., Ren B., Schwager E.H. (2021). Multivariable association discovery in population-scale meta-omics studies. PLoS Comput. Biol..

