# Supplementary material for: Anaerobutyricum soehngenii Reduces Hepatic Lipogenic Pathways and Increases Intestinal Gluconeogenic Gene Expression in Metabolic-Dysfunction-Associated Steatotic Liver Disease (MASLD) Mice
Source: Int J Mol Sci. 2024 Mar 20;25(6):3481. doi: 10.3390/ijms25063481 (PMC10970496; doi:10.3390/ijms25063481)
Supplement: Supplementary file 1 [file ijms-25-03481-s001.zip › ijms-2901660-supplementary.pdf]

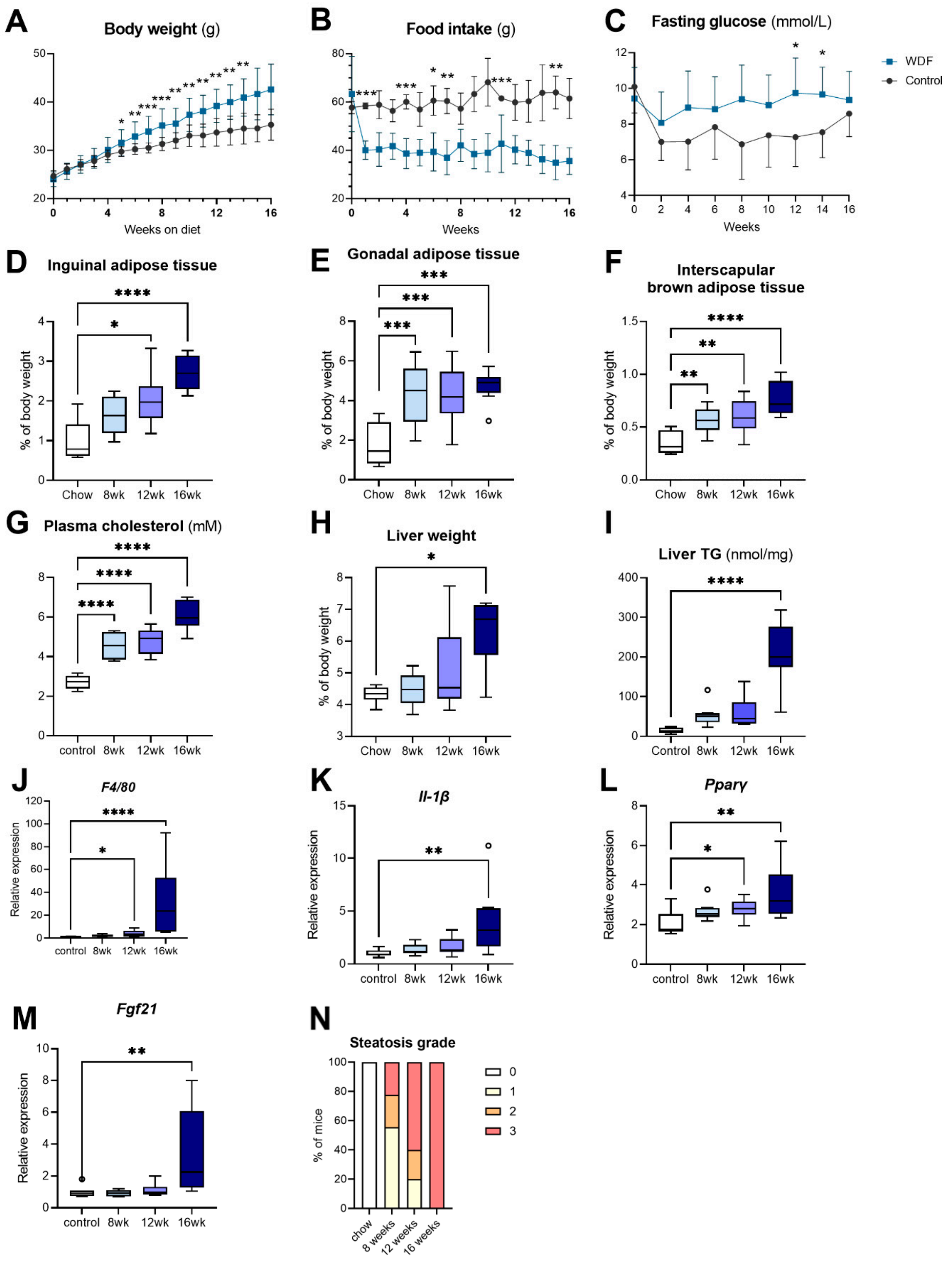

Figure S1: Characteristics of mice fed WDF diet or control chow. (A-C) Body weight, food intake and fasting glucose levels over time; (D-F) Relative adipose tissue weights of mice on the WDF diet for 8, 12 or 16 weeks and chow fed control mice; (G) Plasma cholesterol levels (H-I) Relative liver weight and liver triglyceride content; (J-M) Hepatic gene expression profiles; (N) hepatic steatosis grade. Data shown as boxplots with 25th-75th percentiles and whiskers following Tukey's method, n=8-10 mice per group, *p<0.05; **p<0.01; ***p<0.001; ****p<0.0001 vs control.

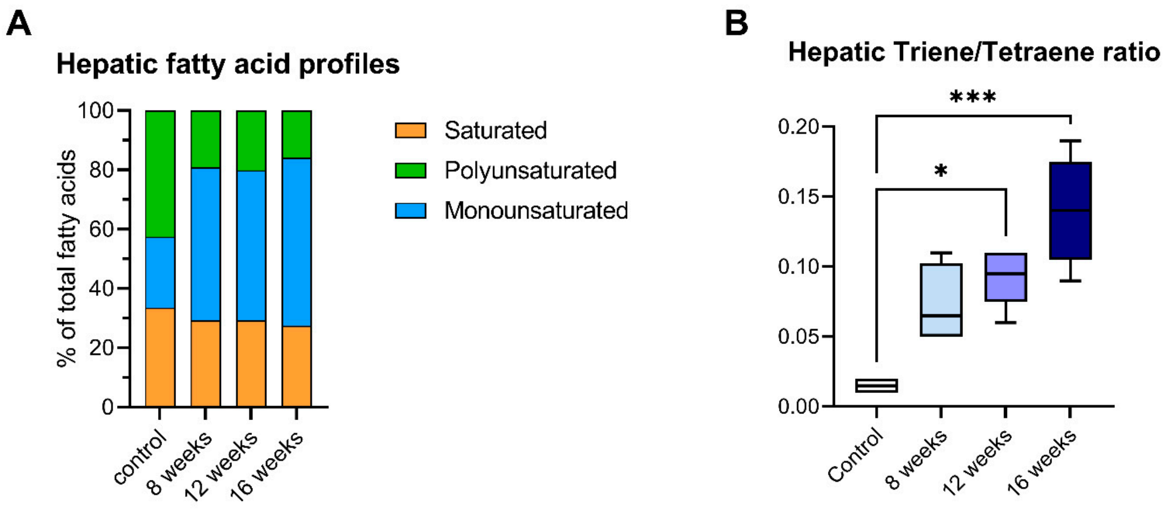

Figure S2: Hepatic fatty acid profiling of mice on the WDF diet for 8, 12 or 16 weeks and chow fed control mice. Relative concentration of total hepatic fatty acid pool is shown. Data shown as boxplots with 25th-75th percentiles and whiskers following Tukey's method n=6 mice per group, *$p<0.05$; ***$p<0.001$ vs control.

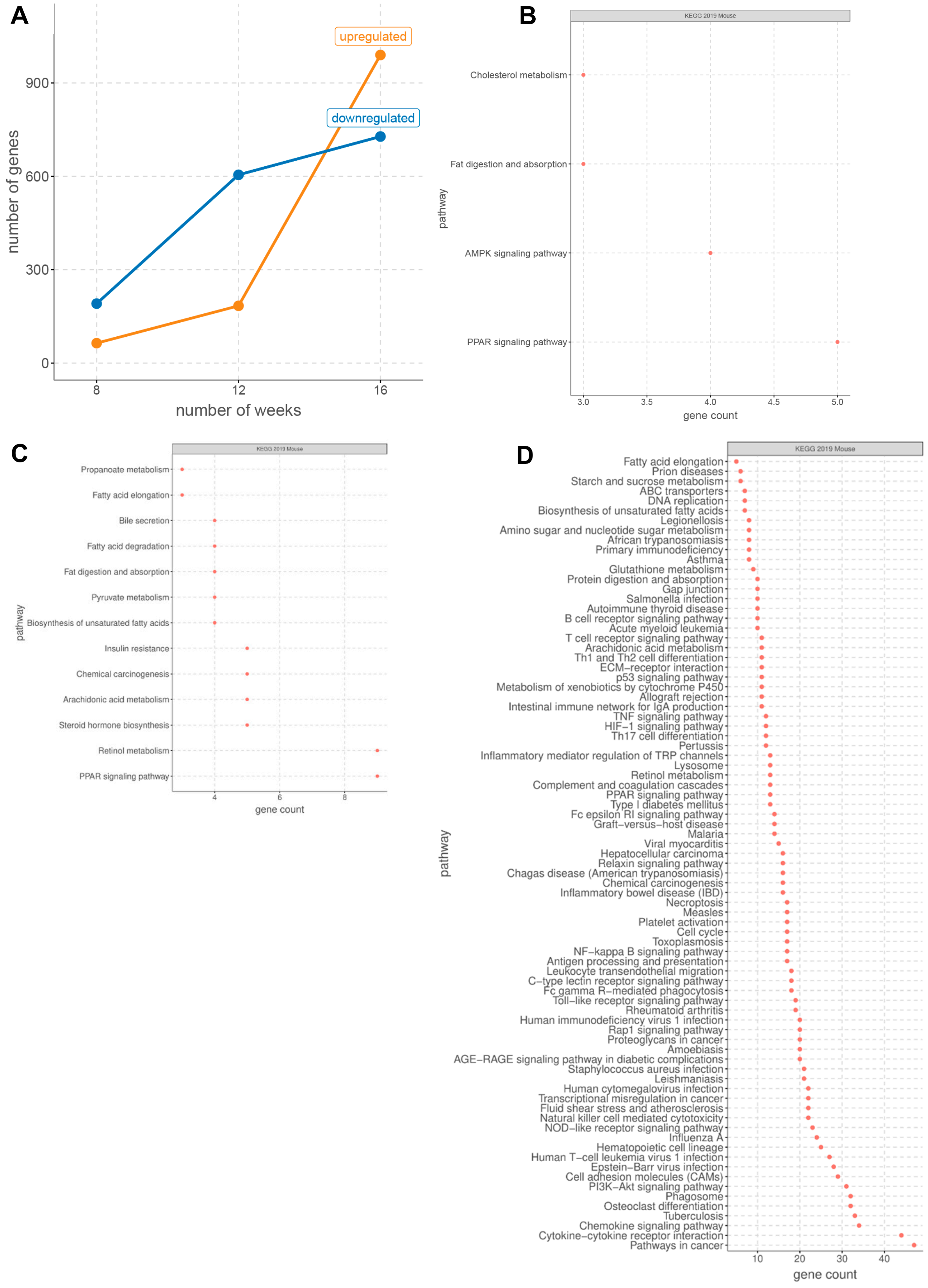

Figure S3: Gene expression alterations over time when mice were fed the WDF diet. (A) Number of genes of which hepatic expression was altered after 8, 12 or 16 week WDF diet; (B) Upregulated pathways after 8 weeks; (C) Upregulated pathways after 12 weeks; (D) Upregulated pathways after 16 weeks.

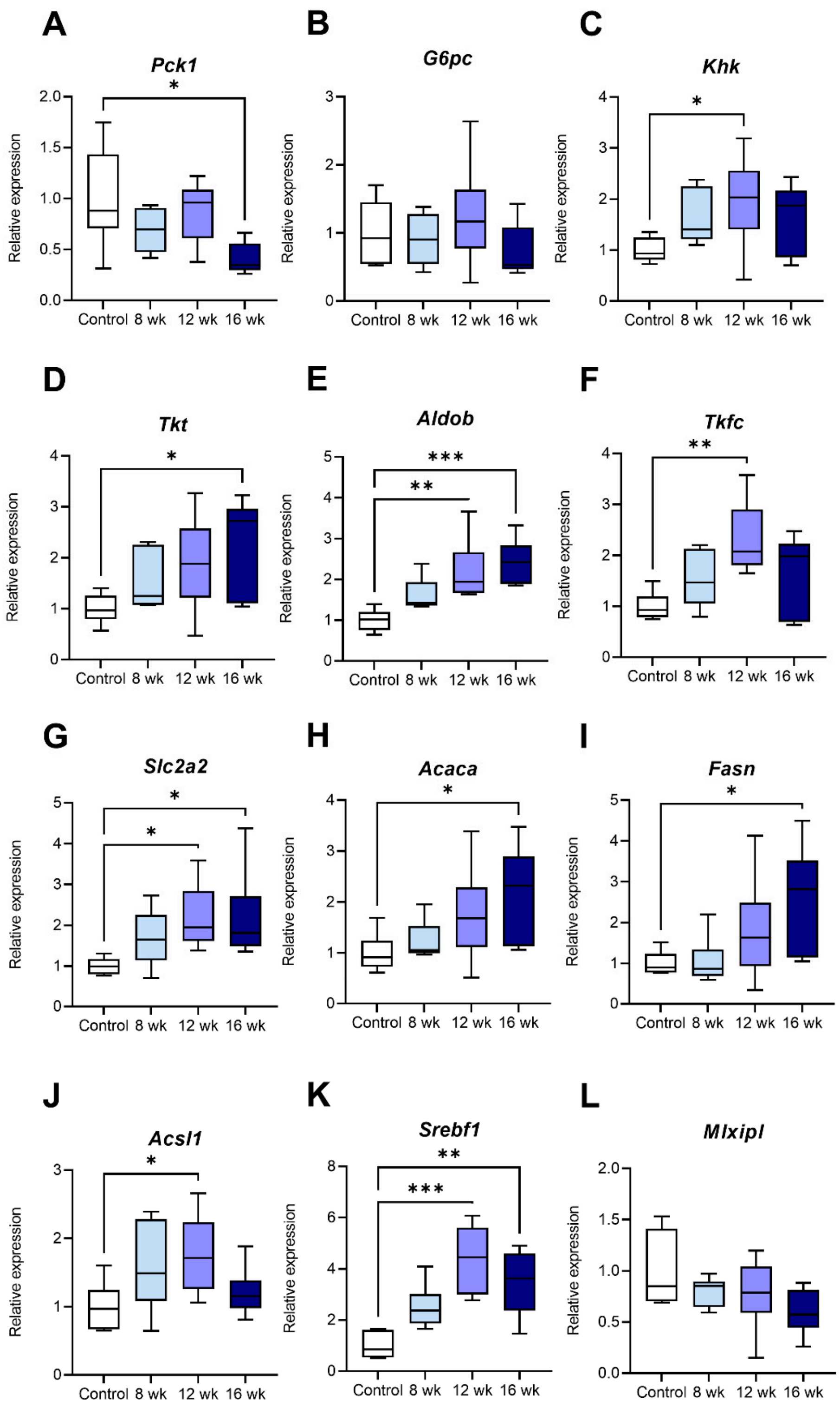

Figure S4: Alterations in hepatic mRNA expression of selected gluconeogenic, fructolytic and lipogenis genes over time when mice were fed the WDF diet. Data shown as boxplots with 25th-75th percentiles and whiskers following Tukey's method, n=6 mice per group. Significant results versus control mice are shown, * p<0.05; **p<0.01; ***p<0.001.

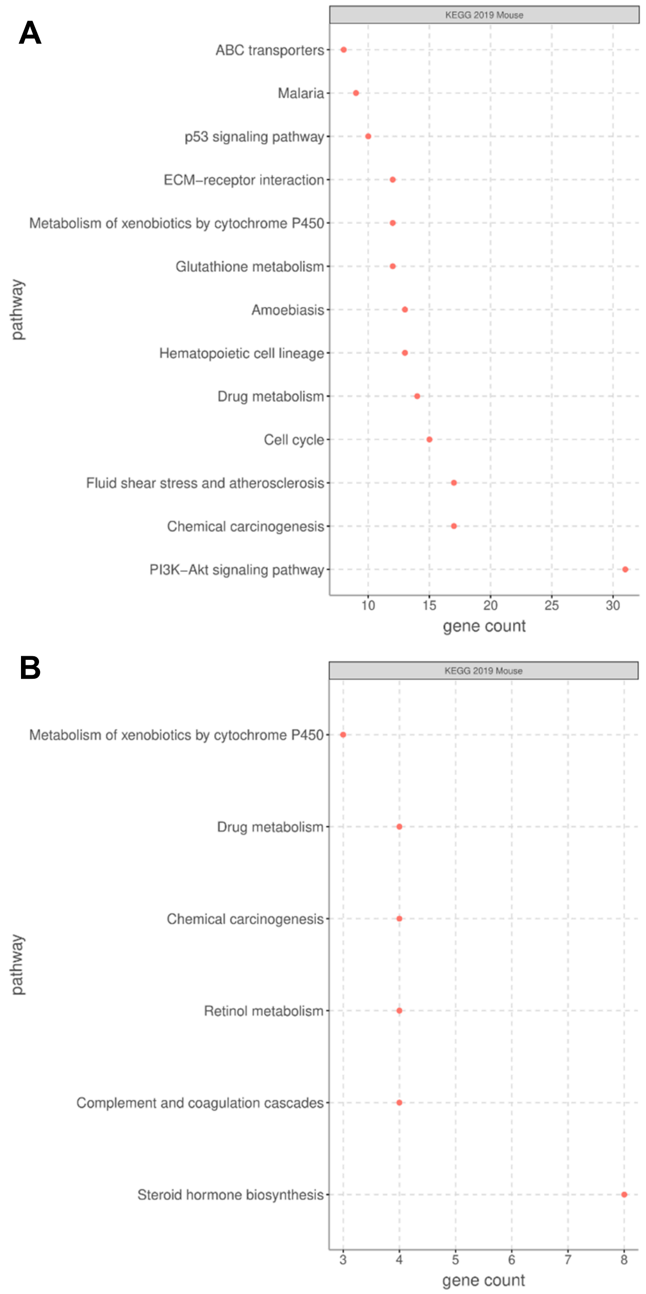

Figure S5: Linearly altered pathways over time on WDF feeding. (A) Upregulated pathways; (B) Downregulated pathways.

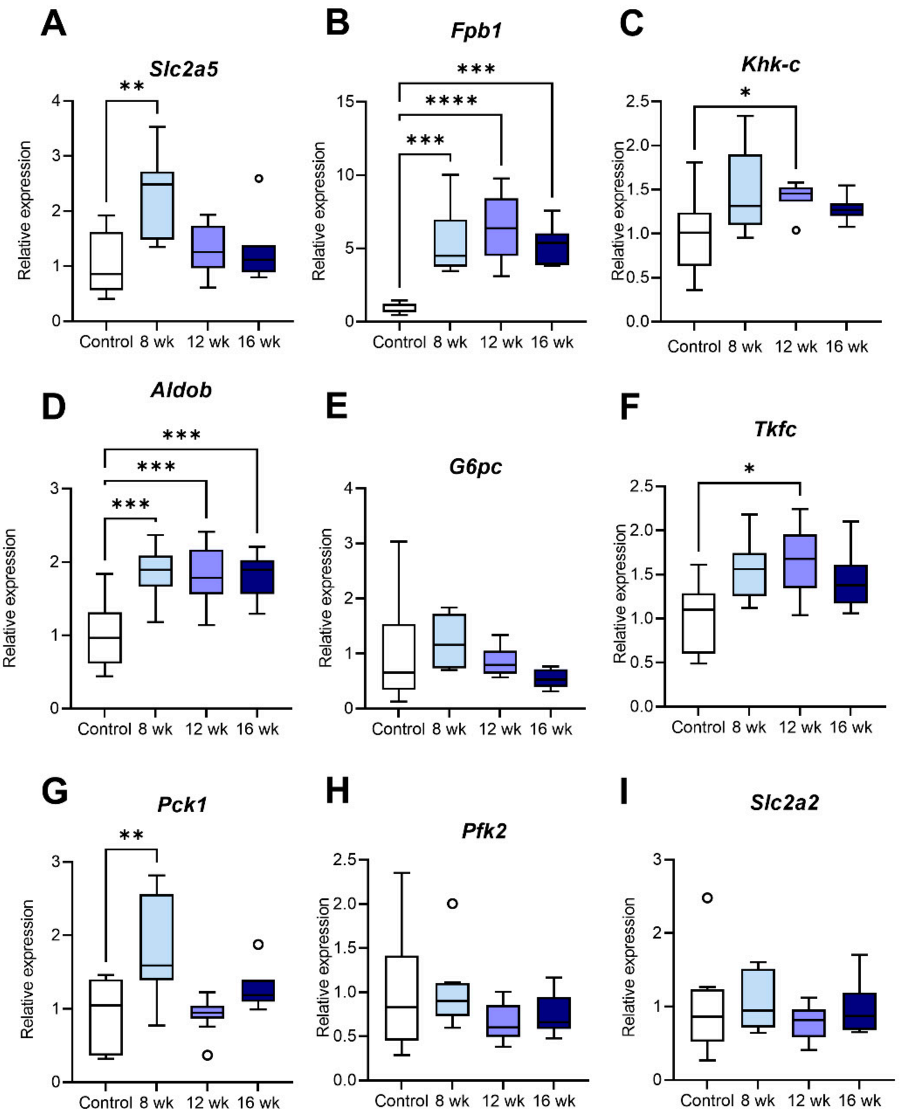

Figure S6: Jejunal expression of fructolysis and gluconeogenic genes over time on WDF. Data shown as boxplots with 25th-75th percentiles and whiskers following Tukey's method, n=6-10 mice per group, $^*p<0.05$; $^{**}p<0.01$; $^{***}p<0.001$; $^{****}p<0.0001$ vs control.

Table S1: Differentially expressed genes between A. soehngenii 10^8 CFU and placebo treated WDF-fed mice

Gene	log2 fold change	Adjusted p-value
Arid5a	1.92942775173682	0.0278631075864381
Chd9	1.29807741475366	0.0278631075864381
Cxcl1	2.37809921413203	0.00223122262252163
Ddit4	2.17779988023803	0.0153403595793546
Fkbp5	1.32821952253346	0.00765976158126705
Gm10095	23.28696963332	7.11338752290865e-05
Gm10359	27.269422318767	3.48268327537329e-07
Gm10718	4.95116369066219	0.0223266229346756
Gm10800	4.54113953188488	0.0153403595793546
Gm10801	5.96626278002467	0.0177328540243074
Gm12671	27.269422318767	3.48268327537329e-07
Gm14440	8.39385877050414	0.00765976158126705
Gm20431	22.8976927737886	0.00025484983067596
Gm21897	2.04311991732719	0.0363967350885441
Gm4294	23.0739441403148	3.48268327537329e-07
Gm47191	4.46808867949633	0.0359110333719121
Igfbp1	2.35859216129421	0.0363967350885441
Il1r1	1.87810567046692	0.000172445651603964
Lpin1	1.81674343649341	0.0139563872644543
Lurap1l	0.685828680524178	0.0139563872644543
Map3k6	1.98470986891677	0.0197570754833017
Mt2	2.97258101436139	0.00849027648447544
Rpl38-ps2	20.5432032443614	0.00549692509091045
Rps13-ps1	21.9241829375954	0.00025484983067596
Slc25a27	0.990892961061582	0.0139563872644543
Taf9	2.39779848240783	0.0411921213203538
Tiparp	1.97507356907475	0.0139563872644543
Vmn1r43	2.48058738576038	0.0139563872644543
Zfp729a	0.522326975771016	0.0278631075864381
Nfe2l2	0.448642176797661	0.0127187565189504

Arid5a = AT-rich interaction domain 5A; Chd9 = Chromodomain helicase DNA binding protein 9; Cxcl1 = C-X-C motif chemokine ligand 1; Ddit4 = DNA-damage-inducible transcript 4; Fkbp5 = FK506 binding protein 5; Igfbp1 = Insulin-like growth factor binding protein 1; Il1r1 = Interleukin 1 receptor, type I; Lpin1 = Lipin1; Lurap1l = Leucine rich adaptor protein 1-like; Map3k6 = Mitogen-activated protein kinase kinase kinase 6; Mt2 = Metallothionein 2; Nfe2l2 = Nuclear factor, erythroid derived 2, like 2; Rpl38-ps2 = Ribosomal protein L38, pseudogene 2; Rps13-ps1 = Ribosomal protein S13, pseudogene 1; Slc25a27 = Solute carrier family 25, member 27; Taf9 = TATA-box binding protein associated factor 9; Tiparp = TCDD-inducible poly(ADP-ribose) polymerase; Vmn1r43 = Vomeronasal 1 receptor 43; Zfp729a = Zinc finger protein 729a. Gene names starting with Gm denote mouse genes (Genus musculus) with unknown function.

Table S2: List of genes that were differentially expressed over time on WDF diet and of which expression was reversed upon A. soehngenii treatment.

Gene name	log2 fold change over time	log2 fold change after treatment
Chd9	-0.540737296100564	1.29807741475366
Cxcl1	-2.05081231907583	2.37809921413203
Fbp1	0.701756708952859	-0.500023569008426
Igfbp1	-1.41718049842732	2.35859216129421
Il1r1	-1.17544069274253	1.87810567046692
Lpin1	-1.71598195523181	1.81674343649341
Map3k6	-0.945973409208895	1.98470986891677
Slc25a27	-1.39981325063274	0.990892961061582
Zfp729a	-0.367008711515888	0.522326975771016

Table S3: Macronutrient compositions of the two used diets.

Nutrient	Control chow	High-fat/high-cholesterol diet
Proteins (%)	16.4	19.8
Carbohydrates (%)	48.5	41.0
Fat (%)	3.7	21.0
Cholesterol (%)	-	0.15
Fiber (%)	18.5	5.0

Table S4: Sequences of used primers.

**Gene**	**Forward primer**	**Reverse primer**
18S (housekeeping gene liver)	CACTTTTGGGGCCTTCGTG	GCAAAGGCCCAGAGACTCATT
36b4 (housekeeping gene liver)	GGACCCGAGAAGACCTCCTT	GCACATCACTCAGAATTTCAATGG
Aldob	AGCGGGCTATGGCTAACTG	GGTGTAGGAGGCTGTGAAGAG
F4/80	TGACAACCAGACGGCTTGTG	GCAGGCGAGGAAAAGATAGTGT
Fbp1	GCATCGCACAGCTCTATGGT	ACACAGGTAGCGTAGGACGA
Fgf21	CTCTCTATGGATCGCCTCACTTT	TGGGCTTCAGACTGGTACAC
G6pc	CCGGTGTTTGAACGTCATCT	CAATGCCTGACAAGACTCCA
HPRT (housekeeping gene jejunum)	TGACACTGGTAAAACAATGCAA	GTCCTTTTCACCAGCAAGCT
Il-1β	GCAACTGTTCCTGAACTCAACT	ATCTTTTGGGGTCCGTCAACT
Khk-c	TGGCAGAGCCAGGGAGAT	ATCTGGCAGGTTCGTGTCGTA
PPARγ	AGGACTGTGTGACAGACAAGA	TGTGTCAACCATGGTAATTTCAGTA
Pck1	ATGTGTGGGCGATGACATT	AACCCGTTTTCTGGGTTGAT
Pfk2	ATGCCACCAATACCACTCGG	GCTCGACACTTTTACCTCCAGA
Slc2a2	AATGGTCGCCTCATTCTTTG	AGCCAACATTGCTTTGATCC
Slc2a5	GGTTGGAATCTGTGCAGGTAT	TGATGAAGAGTTGCGGGACC
TBP (housekeeping gene jejunum)	CTACCGTGAATCTTGGCTGTAAAC	AATCAACGCAGTTGTCCGTGGC
Tkfc	GCCAAGGTCTCTGTGACTGG	TCTGCTTTGATGTTACACCTCCT

18S = 18S ribosomal RNA; 36b4 = Ribosomal protein lateral stalk subunit P0 (Rplp0); Aldob = Aldolase B; F4/80 = Adhesion G protein-coupled receptor E1 (Adgre1); Fbp1 = Fructose bisphosphatase 1; Fgf21 = Fibroblast growth factor 21; G6pc = glucose-6-phosphatase; HPRT = Hypoxanthine phosphoribosyltransferase 1; Il-1β = Interleukin 1 beta; Khk-c = Ketohexokinase-C; PPARγ = Peroxisome proliferator activated receptor gamma; Pck1 = phosphoenolpyruvate carboxykinase 1; Pfk2 = phosphofructokinase-2; Slc2a2 = Solute Carrier Family 2 Member 2 (Glut2); Slc2a5 = Solute Carrier Family 2 Member 5 (Glut5TBP = TATA box binding protein; Tkfc = Triokinase/FMN cyclase.
